# Log *P*_oct_/SA Predicts the Thermoresponsive Behavior of P(DMA-*co*-RA) Statistical Copolymers

**DOI:** 10.1021/acsmacrolett.1c00776

**Published:** 2022-03-22

**Authors:** Irem Akar, Jeffrey C. Foster, Xiyue Leng, Amanda K. Pearce, Robert T. Mathers, Rachel K. O’Reilly

**Affiliations:** † School of Chemistry, University of Birmingham, Edgbaston, Birmingham B15 2TT, United Kingdom; § Department of Chemistry, Pennsylvania State University, New Kensington, Pennsylvania 15068, United States

## Abstract

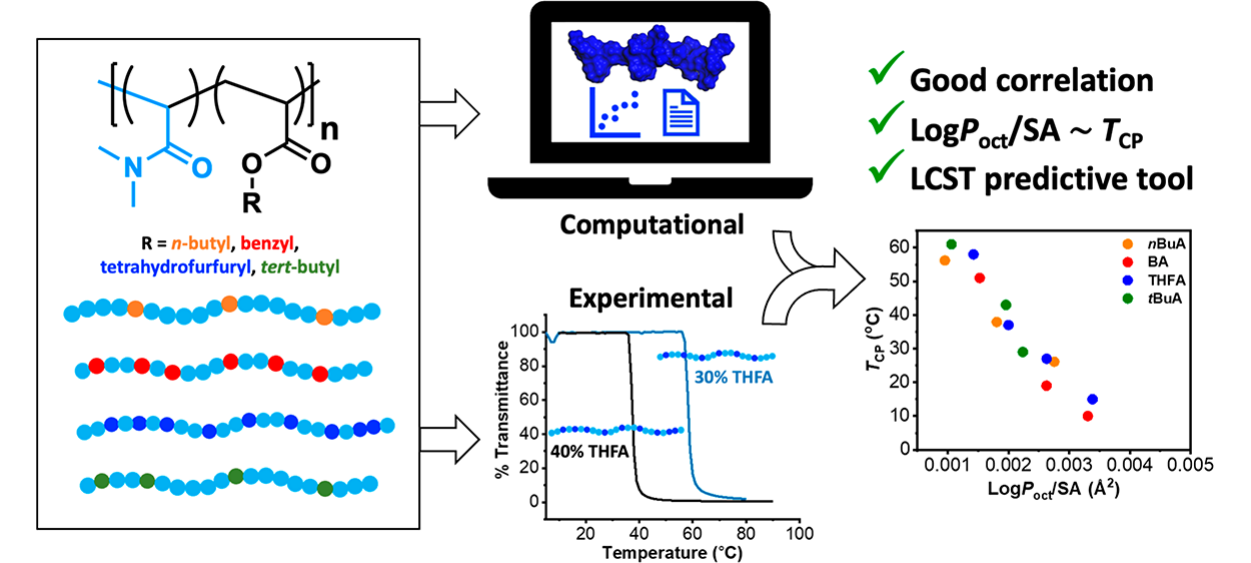

Polymers that exhibit a lower critical solution temperature (LCST) have been of great interest for various biological applications such as drug or gene delivery, controlled release systems, and biosensing. Tuning the LCST behavior through control over polymer composition (e.g., upon copolymerization of monomers with different hydrophobicity) is a widely used method, as the phase transition is greatly affected by the hydrophilic/hydrophobic balance of the copolymers. However, the lack of a general method that relates copolymer hydrophobicity to their temperature response leads to exhaustive experiments when seeking to obtain polymers with desired properties. This is particularly challenging when the target copolymers are comprised of monomers that individually form nonresponsive homopolymers, that is, only when copolymerized do they display thermoresponsive behavior. In this study, we sought to develop a predictive relationship between polymer hydrophobicity and cloud point temperature (*T*_CP_). A series of statistical copolymers were synthesized based on hydrophilic *N*,*N*-dimethyl acrylamide (DMA) and hydrophobic alkyl acrylate monomers, and their hydrophobicity was compared using surface area-normalized octanol/water partition coefficients (Log *P*_oct_/SA). Interestingly, a correlation between the Log *P*_oct_/SA of the copolymers and their *T*_CP_s was observed for the P(DMA-*co*-RA) copolymers, which allowed *T*_CP_ prediction of a demonstrative copolymer P(DMA-*co*-MMA). These results highlight the strong potential of this computational tool to improve the rational design of copolymers with desired temperature responses prior to synthesis.

“Smart” polymers that change their physical or chemical structures upon exposure to external stimuli such as light, pH, redox state, ultrasound, and temperature have been used extensively in a range of applications from biosensors to drug delivery systems.^1−5^ Among these stimuli, temperature has been the most widely studied on account of its easy external applicability and the abundance of methods to tune thermoresponsive behavior within the desired range.^6^ Thermoresponsive polymers display either a lower critical solution temperature (LCST) or an upper critical solution temperature (UCST) behavior in water, where they undergo structural changes and thus changes in their solubility upon heating up above or cooling down below a specific temperature, respectively.^3,7^ In general, LCST-based systems are more preferable than UCST-based systems, particularly for biological applications, because of the high-temperature requirements of the latter.^8,9^ In an LCST-based system, polymers are soluble below their LCST on account of strong interactions between polymer chains and the solvent (water); however, upon heating above a specific temperature, they undergo a phase transition where they become immiscible as a consequence of the weakening of the polymer–solvent interactions.^10^

It is desirable to be able to tune polymer LCST temperatures in order to suit the requirements of a particular application. Several methods have been studied toward this, such as changing the polymer molecular weight, hydrophobicity, or solution concentration.^11−16^ In particular, tuning polymer hydrophobicity is an interesting strategy, as changes to overall hydrophobicity can readily modulate polymer–solvent interactions.^17^ For example, Sumerlin and Vogt reported a method to decrease poly(*N*-isopropylacrylamide) (PNIPAM) LCST by moving from a linear to branched architecture, which increased polymer hydrophobicity through an increase in hydrophobic end groups. They confirmed that the hydrophobic end groups had the greatest impact on polymer LCST (rather than branching) by showing that polymer LCSTs increased significantly upon removal of the end groups.^18^ Another route to tune polymer hydrophobicity is via copolymerization of a high LCST monomer with monomers of lower LCST.^19−24^ For example, Lutz and Hoth reported copolymers of oligoethylene glycol monomethyl ether methacrylate (OEGMA) and diethylene glycol methacrylate (DEGMA) where they showed that the cloud point temperature (*T*_CP_) of the copolymers decreased from 90 to 28 °C as the molar quantity of DEGMA increased.^25^ Additionally, previous work has shown linear correlations of *T*_CP_ to hydrophobic mole fraction for copolymers of *N*-isopropylacrylamide (NiPA) and *N*-isopropylmethacrylamide (NiPMA) and poly(ethylene glycol) monomethyl ether methacrylate (PEGMA) and methyl methacrylate (MMA);^26,27^ however, nonlinear *T*_CP_ behavior has also been reported.^28^ These inconsistencies could be due to differences in experimentation, chain end effects, or the type of polymerization employed. Thus, despite some work in the field to date, there are still fundamental gaps in our understanding of how LCST behavior can be tuned using copolymerization, such as isolating the effect of polymer structure and specific monomer chemistry. Specifically, the ability to generate overall design rules for achieving desired thermoresponsive behavior would contribute essential knowledge toward the design of copolymers for many given applications.

Hydrophobicity is one of the most important phenomena that has been investigated to explain polymer behavior in bulk or solution; however, the influence of polymer hydrophobicity on solution behavior from a theoretical perspective is relatively underexplored.^29^ In medicinal chemistry, hydrophobicity of small molecules can be quantified via octanol–water partition coefficient (Log *P*_oct_) calculations, which describes the partitioning of a substance between octanol and water.^30,31^ Inspired by this, Mathers and co-workers sought to adapt this method to computationally predict the hydrophobicity of macromolecules, developing a surface-area-normalized method (Log *P*_oct_/SA). Subsequent studies have shown that the addition of the surface area normalization improves the predictive power for polymers compared to standard small molecule methods.^32−36^

In our previous study we were interested in correlating the polymer hydrophobicity to its LCST behavior by investigating the relationship between the Log *P*_oct_/SA of a series of statistical copolymers of hydrophilic OEGMA with different hydrophobic methacrylate comonomers (Figure 1A). We aimed to determine a correlation between a polymer hydrophobicity and its *T*_CP_, thereby reducing the experimental workload by predicting the *T*_CP_ of new copolymers prior to synthesis. However, we found that the strongest influence of the copolymer *T*_CP_ was the hydrophobic comonomer mol %, that is, the grafting density rather than the chemical identity of the comonomers, as the brushy nature of the OEGMA dominated the phase transition.^21^ This finding inspired us to investigate whether a correlation could be found between polymer hydrophobicity and the *T*_CP_ when using nonbrushy monomers; thus, providing a route to uniquely tune polymer LCST behavior using specific monomer chemistry (Figure 1B).

**Figure 1:**
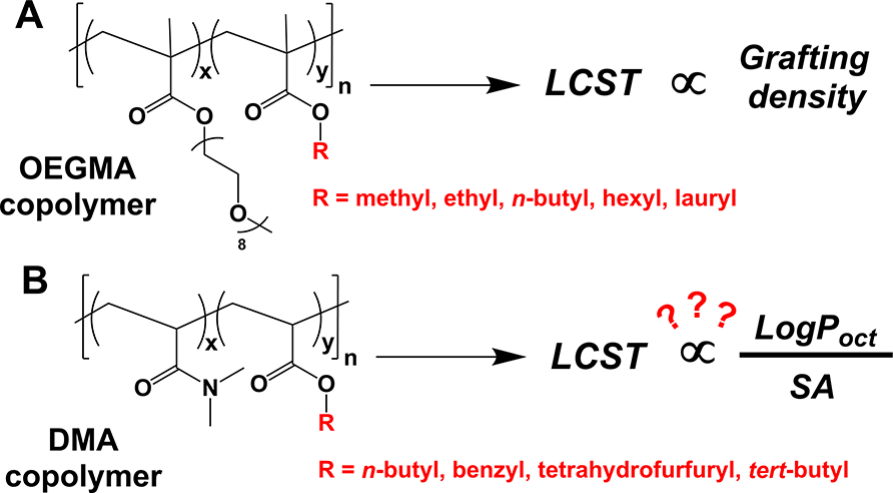
Our studies on how hydrophobicity influences thermoresponsive behavior of (A) brushy polymers and (B) nonbrushy polymers.

With the goal of realizing this, we selected hydrophilic *N*,*N*-dimethyl acrylamide (DMA) as our nonbrushy monomer of interest, due to its simple chain structure and commercial availability, and alkyl acrylates such as *n*-butyl acrylate (*n*BuA), benzyl acrylate (BA), tetrahydrofurfuryl acrylate (THFA), and *tert*-butyl acrylate (*t*BuA) for the hydrophobic component on account of their commercial availability and compatibility with polymerization conditions. Interestingly, to the best of our knowledge, no literature studies report DMA homopolymers displaying LCST behaviors under the dilute conditions that are typically employed during *T*_CP_ measurements (ca. 1–10 mg mL^–1^ polymer), with only one study by Fischer et al. reporting a DMA homopolymer with a very high *T*_CP_ at a solution concentration of 20 mg mL^–1^.^37^ Thus, our aim was two-fold: to not only establish correlations for polymer hydrophobicity and *T*_CP_, but to investigate the intriguing LCST behavior of this largely nonthermoresponsive monomer at lower concentrations and temperature windows.

To this end, we initially synthesized a library of copolymers based on DMA and various hydrophobic alkyl acrylates (RA, R = *n*-butyl, benzyl, tetrahydrofurfuryl, and *tert*-butyl) in order to observe the effect of monomer chemistry and copolymer composition and studied their LCST response. *T*_CP_ was used as a proxy of the LCST behavior as it is a macroscopic effect that can be detected easily via dynamic light scattering,^38^ differential scanning calorimetry,^39^ microdifferential scanning calorimetry,^21^ and UV–vis spectroscopy.^21^ Then, we attempted to correlate the *T*_CP_ of the copolymers to their hydrophobicity, which was determined by calculating the Log *P*_oct_/SA of oligomeric models representative of the final copolymers. Overall, copolymer MW and the targeted hydrophobic mol % were maintained as consistently as possible across each series. The copolymers were prepared via reversible addition–fragmentation chain transfer (RAFT) polymerization in 1,4-dioxane for 4 h until targeted DPs were reached (Figure 2A). The final molar composition of the purified copolymers was determined using ^1^H NMR spectroscopy by relative integration of resonances corresponding to each monomer (Figures 2B and S1–S4). Kinetic analysis showed that both DMA and RA monomers were consumed at an approximately equal rate, confirming the statistical nature of the copolymerizations (Figures S5–S8). Molecular weight distributions (MWDs) for the P(DMA-*co*-RA) copolymers were determined using size-exclusion chromatography (SEC). Copolymers were obtained with narrow and symmetrical MWDs (Figures 2D and S6–S8). Variations in number-average MW (*M*_n_) and dispersity (*Đ*_M_) values were determined by calculating the coefficients of variance. Using this measure, *M*_n_ varied by only 2% across the entire data set, while *Đ*_M_ varied by 0.04% with all values <1.36. Turbidity measurements were conducted using UV–vis spectroscopy in order to measure the *T*_CP_ of the copolymers. Changes in the percentage transmittance were recorded at λ = 550 nm within the temperature range of 0 to 90 °C. Temperature points that correspond to 50% transmittance values were taken as the *T*_CP_ of polymers (see Supporting Information for a detailed method). In general, an inverse relationship was observed between the *T*_CP_ of P(DMA-*co*-RA) copolymers and the RA content (Figures 2E and S9).

**Figure 2:**
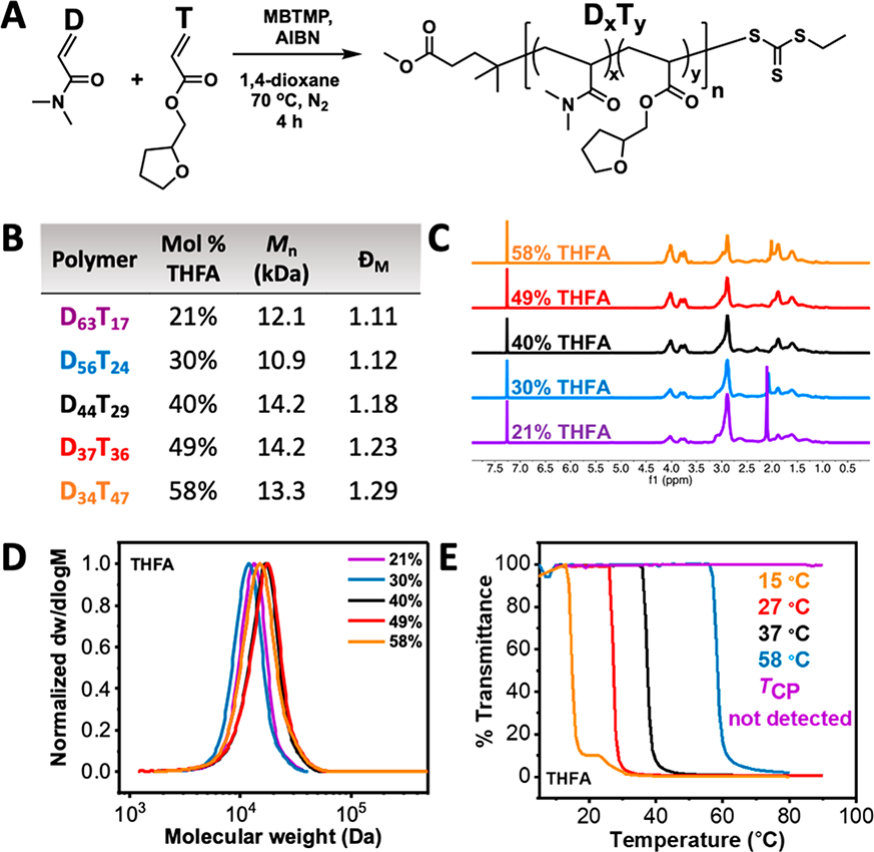
(A) Synthetic scheme for the preparation of P(DMA-*co*-RA) statistical copolymers. THFA is used as the comonomer in this example. (B) Molar composition (determined by ^1^H NMR spectroscopy), number-average MW (*M*_n_), and dispersity (*Đ*_M_; determined by SEC) of P(DMA-*co*-THFA) copolymers. (C) ^1^H NMR spectra of P(DMA-*co*-THFA) copolymers in CDCl_3_ (300 MHz). (D) Normalized SEC molecular weight distributions for the P(DMA-*co*-THFA) series (eluent: CHCl_3_ + 0.5 v/v% NEt_3_, PMMA standards). (E) Percent transmittance as a function of temperature for the P(DMA-*co*-THFA) copolymers dissolved in H_2_O at 10 mg/mL as measured by UV–vis spectroscopy (λ = 550 nm, 0–90 °C, 1 °C min^–1^).

Log *P*_oct_ values of short oligomer models that represent each P(DMA-*co*-RA) copolymer were calculated (at *T* = 298 K) for quantifying hydrophobicity. Then, the Log *P*_oct_ value of each representative model was normalized by surface area using Molecular Dynamics (MD) simulations (see Supporting Information for a detailed model). Normalizing a thermodynamic parameter (i.e., Log *P*_oct_) with a structural parameter (i.e., SA) facilitates comparison of architectural differences resulting from monomer size and functionality as well as oligomer length.^36,40,41^ Furthermore, this strategy conforms to the Lum–Chandler–Weeks theory, which states that above a critical length scale, hydrophobicity scales with surface area rather than volume.^29,42^ Consequently, Log *P*_oct_/SA values provide a general method for correlating hydrophobicity to *T*_CP_ that accommodates models with enough repeating units to represent the actual polymer. Figure 3A shows the repeating units for each of the P(DMA-*co*-RA) copolymers that were used to build oligomer models to calculate the Log *P*_oct_/SA values. The length of the oligomer models varied between 17 and 27 units and the models were built based on the hydrophobic mol % that each copolymer contains (see Supporting Information for detailed model). Log *P*_oct_/SA increased as the hydrophobic mol % in the copolymers increased, confirming the relationship between hydrophobicity and Log *P*_oct_ (Figure 3B).^36^ Comonomers *n*BuA, BA, and *t*BuA produced copolymers with similar slopes, while P(DMA-*co*-THFA) copolymers differed from the others. The ether oxygen in the tetrahydrofuran ring of THFA had a significant influence on Log *P*_oct_/SA values. Importantly, this data demonstrates that the comonomer chemistry plays an important role in overall hydrophobicity of P(DMA-*co*-RA) copolymers. Figure 3C shows the inverse linear relationships between the hydrophobic mol % and *T*_CP_ of the copolymers for each series. Linear regression data for each series is shown in Table S3. This clearly illustrates that the increase in the hydrophobic comonomer content results in an increase in the overall copolymer hydrophobicity, causing the copolymer *T*_CP_ to decrease. Motivated by this, we next plotted the calculated Log *P*_oct_/SA values against the measured *T*_CP_s in order to see if any correlation could be built. Figure 3D shows the inverse relationship between the Log *P*_oct_/SA (polymer hydrophobicity) and the *T*_CP_ of P(DMA-*co*-RA) copolymers with each series possessing a similar slope. This indicates that copolymer hydrophobicity can be directly correlated to its *T*_CP_ for these nonbrushy copolymers, unlike the OEGMA-based brushy copolymers we studied in our previous work. Of importance, the data shown in Figure 3D was fitted using linear, exponential, and polynomial fits. We found that the prediction capability of the linear fit was superior to the polynomial fit and very similar to the exponential fit in terms of the similarity of the measured and calculated *T*_CP_ of the P(DMA-*co*-RA) copolymers. Therefore, we chose the linear fit for the *T*_CP_ prediction due to its greater simplicity. Comparison of the measured and predicted *T*_CP_ for P(DMA-*co*-RA) copolymers showed a reasonably strong correlation, suggesting that this tool could be used for predicting the *T*_CP_ of new nonbrushy copolymers (Figure 3E). Thus, we suggest that the experimental *T*_CP_ of new copolymers can be reliably predicted using this computational method.

**Figure 3:**
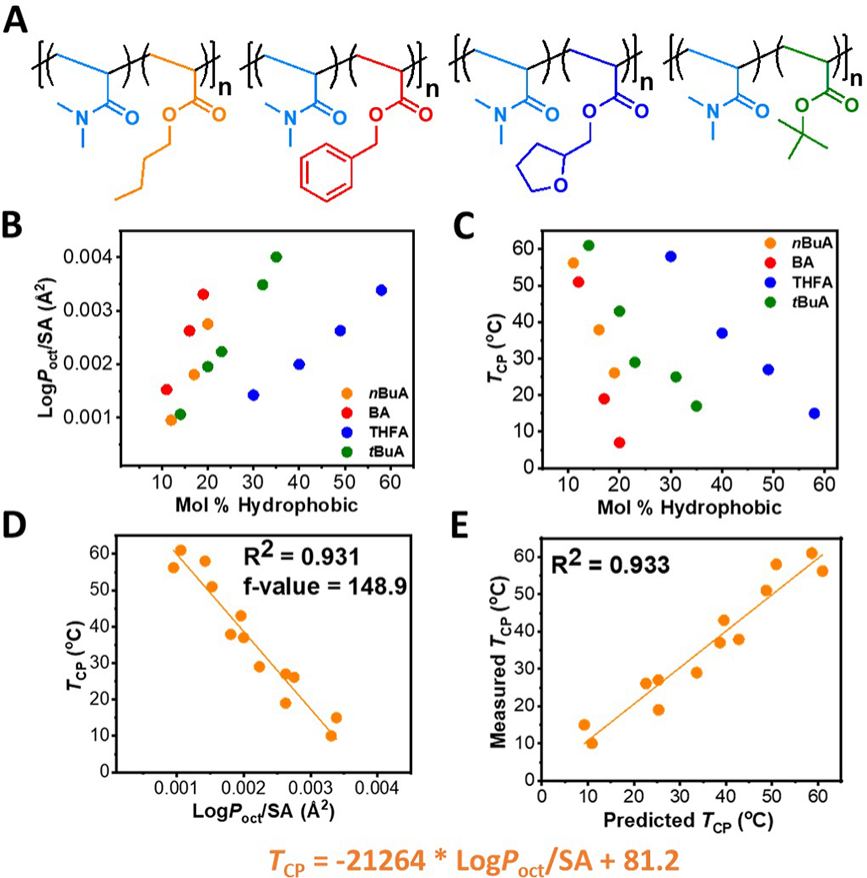
(A) Chemical structures of the repeating units for the P(DMA-*co*-*n*BuA), P(DMA-*co*-BA), P(DMA-*co*-THFA), and P(DMA-*co*-*t*BuA) copolymers, respectively. (B) Calculated Log *P*_oct_/SA values for P(DMA-*co*-RA) copolymer oligomers as a function of the mol % of the hydrophobic comonomer. (C) Plot of *T*_CP_ as measured by UV–vis spectroscopy vs the mol % of hydrophobic comonomer. (D) Plot of *T*_CP_ as measured by UV–vis spectroscopy vs the calculated Log *P*_oct_/SA values for P(DMA-*co*-RA) copolymer oligomers. The solid line represents a linear fit of these data. (E) Comparison between measured *T*_CP_ values of P(DMA-*co*-RA) copolymers and those predicted from their Log *P*_oct_/SA. The solid line represents a linear fit of these data. The equation was generated using the linear fit of the data in the plot of Figure 3D.

It is important to note the significance of these findings in the context of facilitating the targeted design of new copolymers based on monomers known to produce nonresponsive homopolymers. In such cases, conventional methods like the Flory–Fox equation, which determines the thermal properties of polymers based on both weight fraction and thermoresponse of the two homopolymers, cannot be used. Therefore, the predictive tool developed in this work significantly increases ease of access to new thermoresponsive copolymers with varied chemistries and tunable on-demand temperature responses.

Finally, to prove this hypothesis, we chose to design a new copolymer of DMA and the well-known hydrophobic monomer methyl methacrylate (MMA), which was one of the hydrophobic comonomers used in our previous work.

Log *P*_oct_/SA of the P(DMA-*co*-MMA) copolymer with 31% hydrophobic mol % was calculated prior to synthesis, giving a predicted *T*_CP_ of 41 °C when using the equation generated based on the relationship between Log *P*_oct_/SA and measured *T*_CP_. Following the polymer synthesis, the measured *T*_CP_ of the copolymer was determined as 42 °C using UV–vis spectroscopy. This confirmed that the *T*_CP_ of new copolymers could be predicted using this guidance with only minor deviations from the targeted *T*_CP_. Interestingly, unlike the P(DMA-*co*-RA) polymerizations, the copolymerization of DMA and MMA yielded a copolymer with a gradient topology (Figure S10). Based on the fact that Log *P*_oct_/SA could still predict *T*_CP_ for this copolymer, it was hypothesized that the exact copolymer sequence may not be a critical determinant of thermoresponsiveness. Further investigation is warranted to test this hypothesis.

To conclude, we report the synthesis of a series of thermoresponsive P(DMA-*co*-RA) copolymers via copolymerization of DMA and different alkyl acrylate monomers and the investigation of their LCST behavior by measuring the copolymer *T*_CP_s using UV–vis spectroscopy. Analysis of our experimental data using computational modeling of Log *P*_oct_/SA revealed that the thermoresponsive behavior of nonbrushy P(DMA-*co*-RA) copolymers could be related to their hydrophobicity. We validated this method by predicting the *T*_CP_ of a P(DMA-*co*-MMA), which showed good correlation with the experimentally measured *T*_CP_ (1 °C difference from targeted *T*_CP_). Overall, this study demonstrates the strength of the Log *P*_oct_/SA computational modeling tool for the prediction of copolymer interactions in solution. We envisage this to be particularly powerful in the study of thermoresponsive copolymers comprised of monomers that produce nonresponsive homopolymers, thus, widening access to new monomer chemistries that can be used in the rational design of polymers with thermoresponsive behavior.

## Supporting Information Available

Materials, characterization techniques, experimental procedures, and additional data (SEC, ^1^H NMR, and UV–vis spectra) (PDF)
