# Supplementary material for: Log Poct/SA Predicts the Thermoresponsive Behavior of P(DMA-co-RA) Statistical Copolymers
Source: ACS Macro Lett. 2022 Mar 22;11(4):498–503. doi: 10.1021/acsmacrolett.1c00776 (PMC9022432; doi:10.1021/acsmacrolett.1c00776)
Supplement: Supplementary file 1 — mz1c00776_si_001.pdf [file mz1c00776_si_001.pdf]

Supporting Information

"LogP_{oct}/SA predicts thermoresponsive behavior of P(DMA-*co*-RA) statistical copolymers"

Irem Akar,† Jeffrey C. Foster,† Xiyue Leng,† Amanda K. Pearce, † Robert T. Mathers,,§ and Rachel K. O'Reilly*,†*

[†]School of Chemistry, University of Birmingham, Edgbaston, Birmingham, B15 2TT, UK

[§]Department of Chemistry, Pennsylvania State University, New Kensington, PA 15068, USA

*Corresponding Authors: rtm11@psu.edu (R.T.M.), and r.oreilly@bham.ac.uk (R.K.O.R.)

Materials and Methods

Materials

Benzyl acrylate (BA, Alfa-Aesar, 97%), *n*-butyl acrylate (*n*BA, Sigma-Aldrich, 99%), tetrahydrofurfuryl acrylate (THFA, Sigma-Aldrich), *tert*-butyl acrylate (*t*BA, Sigma-Aldrich, 98%), *N,N*-dimethyl acrylamide (DMA, Sigma-Aldrich, 99%), methyl methacrylate (MMA, Sigma-Aldrich, 99%), and 1,4-dioxane (Sigma-Aldrich, 99.8%) were filtered through basic alumina prior to use. 2,2′-Azobis(2-methylpropionitrile) (AIBN) was received from Molekula, recrystallized from methanol and stored at 4 °C. Methyl 2-(butylthio-carbonothioylthio)-2-methylpropanoate (MBTMP) was prepared according to a previously described procedure.[1]

Characterization Techniques

NMR Spectroscopy. ^{1}H-NMR and ^{13}C-NMR spectra were recorded at 300 MHz on a Bruker DPX-300 spectrometer, using chloroform-*d* ($CDCl_3$) as the solvent. Chemical shifts of protons are reported as δ in parts per million (ppm) and are relative to solvent residual peaks.

Size Exclusion Chromatography. Size exclusion chromatography (SEC) analysis was performed on a system composed of an Agilent 1260 Infinity II LC system equipped with an Agilent guard column (PLGel 5 μM, 50 × 7.5 mm) and two Agilent Mixed-C columns (PLGel 5 μM, 300 × 7.5 mm). The mobile phase used was $CHCl_3$ (HPLC grade) containing 0.5% v/v NEt_3 at 40 °C at flow rate of 1.0 mL min^{-1} (polymethyl methacrylate (PM) standards were used for calibration). Detection was conducted using a differential refractive index (RI) detector. Number-average molecular weights (M_n), weight-average molecular weights (M_w) and dispersities ($Đ_M = M_w/M_n$) were determined using the Agilent GPC/SEC software.

UV-Vis Spectroscopy. UV-Vis analysis was performed on an Agilent Cary 3500 UV-Vis Spectrophotometer to record the LCST behavior and temperature dependent transmittance spectra of polymers by heating the samples in solution from 0 ^{o}C to 90 ^{o}C at a rate of 1 ^{o}C min^{-1}. Samples were corrected against a background of DI water. Measurements were carried out using glass cuvettes with a path length of 10.00 mm.

Synthetic Methods

Synthesis of P(DMA-*co*-RA) copolymers

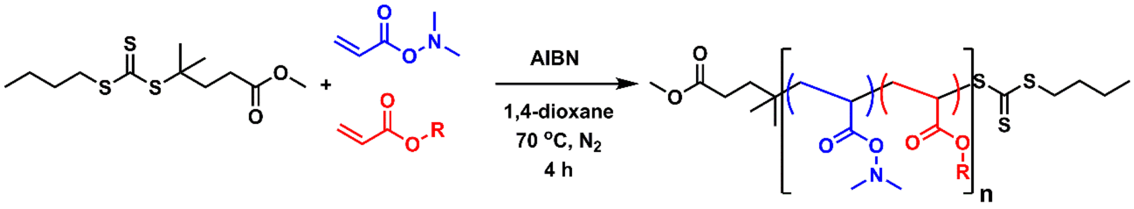

Copolymerization of RA monomers with DMA was conducted using AIBN as the initiator, MBTMP as the RAFT agent, 1,3,5-trioxane as an internal reference, and 1,4-dioxane as the solvent. RA content was varied between 10-20% for *n*BuA, 10-20% for BA, 30-60% for THFA, and 15-25% for *t*BuA, targeting the total DP of 80 to 85 with a molar ratio of [Monomer]/[1,3,5-Trioxane]/[MCETP]/[AIBN] = 85:20:1:0.1 in 1,4-dioxane (monomer/1,4-dioxane = 1:2 by volume). For the synthesis of a copolymer with 20% RA: a stock solution of 10 mg mL^{-1} AIBN was prepared in 1,4-dioxane and 62 µL of this solution (0.6 mg, 3.8 x 10^{-3} mmol, 0.1 eq) was added to a vial of MBTMP (10 mg, 3.8 x 10^{-2} mmol, 1.0 eq), RA monomer (0.6 mmol, 17 eq), DMA (0.26 mL, 2.6 mmol, 68 eq), and 1,3,5-trioxane (68 mg, 7.5 x 10^{-1} mmol, 20 eq) in 1,4-dioxane and mixed in an oven-dried ampoule containing a magnetic stir bar until all solids dissolved. The resulting solution was degassed using at least three freeze-pump-thaw cycles, back-filled with N_2, and placed in a preheated oil bath at 70 °C. Samples were taken periodically to determine the overall and relative conversion of comonomers. Purification was achieved by dialysis (6-8 kDa MWCO) against nanopure water followed by lyophilisation.

Experimental Methods

Copolymerization kinetic studies. Kinetic experiments for one polymer from each series (10% *n*BuA for P(DMA-*co*-*n*BuA), 20% BA P(DMA-*co*-BA), 30% THFA P(DMA-*co*-THFA), and 23% *t*BuA for P(DMA-*co*-*t*BuA) were carried out in order to determine the conversion of each monomer at the same time intervals (i.e., 60, 90, 120, 150, and 180 min). Samples were periodically taken from the polymerization mixtures under nitrogen and then diluted with $CDCl_3$ prior to ^1H NMR analysis. Integrations of monomer signals were compared to those internal standard resonances to determine monomer conversions.

Determination of T_{CP} by UV-Vis spectroscopy. Solutions of P(DMA-*co*-RA) copolymers were prepared at 10 mg mL^{-1} in nanopure water. The temperature-dependent change in transmittance % was recorded at λ = 550 nm within the temperature range of 0 ^oC to 90 ^oC at a heating rate of 1 ^oC min^{-1}. Samples were run for two heating/cooling cycles and T_{CP} values taken as the temperature at which the sample transmittance had decreased to 50% on the second heating cycle.

Evaluation of Oligomer Hydrophobicity

LogP_{oct} Analysis. Octanol-water partition coefficients (LogP_{oct}) were calculated for monomers and oligomeric models in Materials Studio 2020,[2] using an atom-based approach (ALogP98 method)[3] for all molecular models containing C, H, N, and O atoms.

Surface Area Analysis. Octanol-water partition coefficients (LogP_{oct}) were normalized by solvent accessible surface area (SA) using Materials Studio 2020.[4-7] First, single oligomers were subjected to a Geometry Optimization procedure using the Forcite Molecular Dynamics (MD) module with a COMPASS II force field. The force field contains information on important parameters, like preferred bond lengths, bond angles, torsion angles, partial charges, and van der Waals radii that influence the conformation.[8] To minimize energy and determine a preferred conformation, these simulations ran until the energy of the oligomer decreased below predetermined convergence criteria (1×10^{-4} kcal mol^{-1} energy convergence, 0.005 kcal mol^{-1} /Å force convergence, and 5×10^{-5} Å displacement convergence). Second, these SA values represent the Connolly surface area created by an algorithm that rolls a ball over the surface of the oligomer. To ensure the SA values are meaningful in the context of octanol-water partition coefficients (LogP$_{oct}$), the probe had a 1.4 Å radius to match the size of a water molecule. Third, to monitor variations in surface area calculations as the n-mer size increased, oligomers were annealed for 200 cycles using a sinusoidal temperature ramp (300 – 700 K) to maximize variability in SA values. After averaging SA values for cycles 100, 150 and 200, the standard deviation ranged from 0.4-2.7% with an average of 1.2%.

Models. Oligomeric models contained appropriate ratios of DMA and alkyl acrylate units to mimic experimental conditions. In addition, models were proton terminated and contained DMA units randomly distributed throughout the oligomer. For example, *n*BuA/DMA oligomers contained 16-17 DMA units with 2-4 *n*BuA units for a total of 19-20 units such that the amount of *n*BuA ranged from 10.7-19.3 mol %. The quantity of monomers in BA/DMA oligomers ranged from 15-19 DMA units and 2-5 BA units for a total of 17-24 units with 11.8-20.8 mol % BA. THFA/DMA oligomers contained 8-16 DMA units with 4-13 THFA units for a total of 19-27 monomer units to achieve 20.0-57.9 mol % THFA. *t*BuA/DMA oligomers combined 16-18 DMA units with 3-5 *t*BuA units for a total of 20-22 units such that the amount of *t*BuA ranged from 14.3-22.7 mol %.

Supplementary Characterization Data for DMA-*co*-RA Copolymers

Table S1. Molecular characteristics of P(DMA-*co*-RA) copolymers prepared *via* RAFT, as determined by ^{1}H NMR spectroscopy and SEC analyses.[a]

Polymer	Target RA Percentage	Final RA Percentage[b]	DP[c]	$M_{n,\,NMR}$[c] (kDa)	$M_{n,\,SEC}$[d] (kDa)	$Đ_M$[e]
**P(DMA-*co*-*n*BuA)**	10	11	84	8.9	10.5	1.10
	15	15	84	9.0	10.7	1.15
	20	19	83	9.0	12.1	1.11
**P(DMA-*co*-BA)**	10	12	84	9.2	9.6	1.21
	15	17	82	9.3	10.4	1.13
	20	20	83	9.6	11.0	1.13
**P(DMA-*co*-THFA)**	20	21	80	9.0	12.1	1.12
	30	30	80	9.6	10.9	1.12
	40	40	73	9.2	14.2	1.18
	50	49	73	9.6	14.2	1.23
	60	58	81	11.0	13.3	1.29
**P(DMA-*co*-*t*BuA)**	15	14	83	8.8	13.4	1.35
	20	20	83	9.0	10.4	1.09
	25	23	83	9.0	11.4	1.10
**P(DMA-*co*-MMA)**	30	31	81	8.3	12.9	1.93

[a] Conditions – [Monomer]/[1,3,5-Trioxane]/[MBTMP]/[AIBN] = 85:20:1:0.1 in 1,4-dioxane (monomer/1,4-dioxane = 1 : 2 by volume).
[b] Determined by ^{1}H NMR spectroscopy ($CDCl_3$, 300 MHz).
[c] Calculated from monomer conversion.
[d] M_n and $Đ_M$ determined by SEC (in $CHCl_3$ + 0.5% NEt_3, calibrated against PMMA standards).

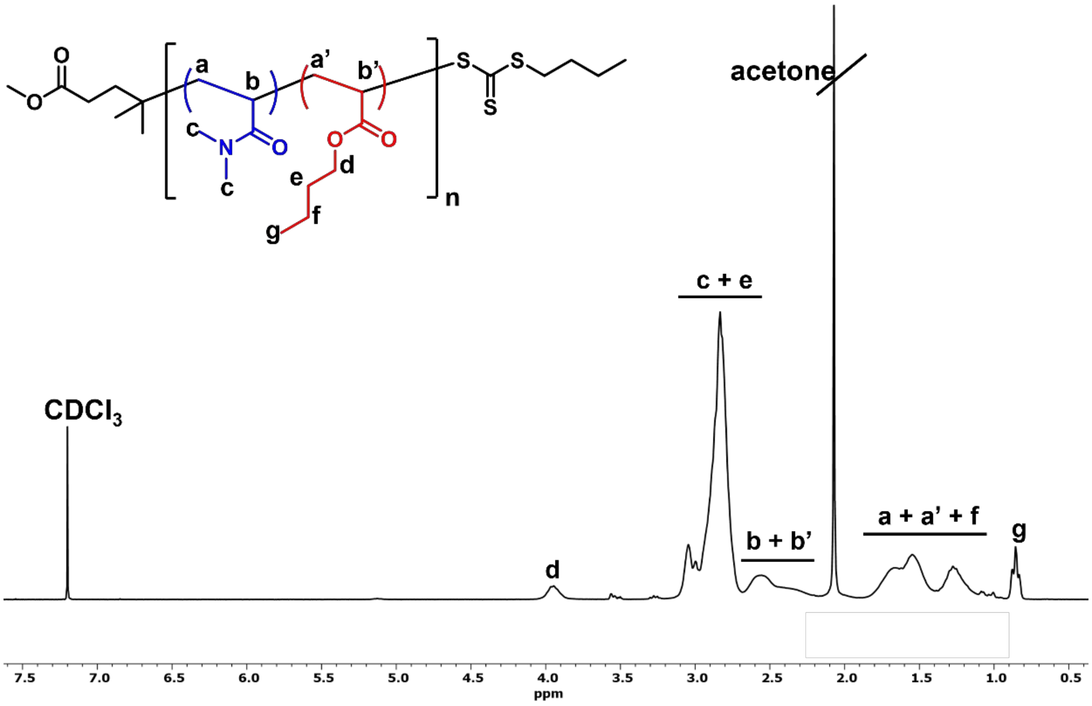

Figure S1. ^{1}H-NMR spectrum of P(DMA-*co*-*n*BuA)$_{84}$ in $CDCl_3$ (300 MHz).

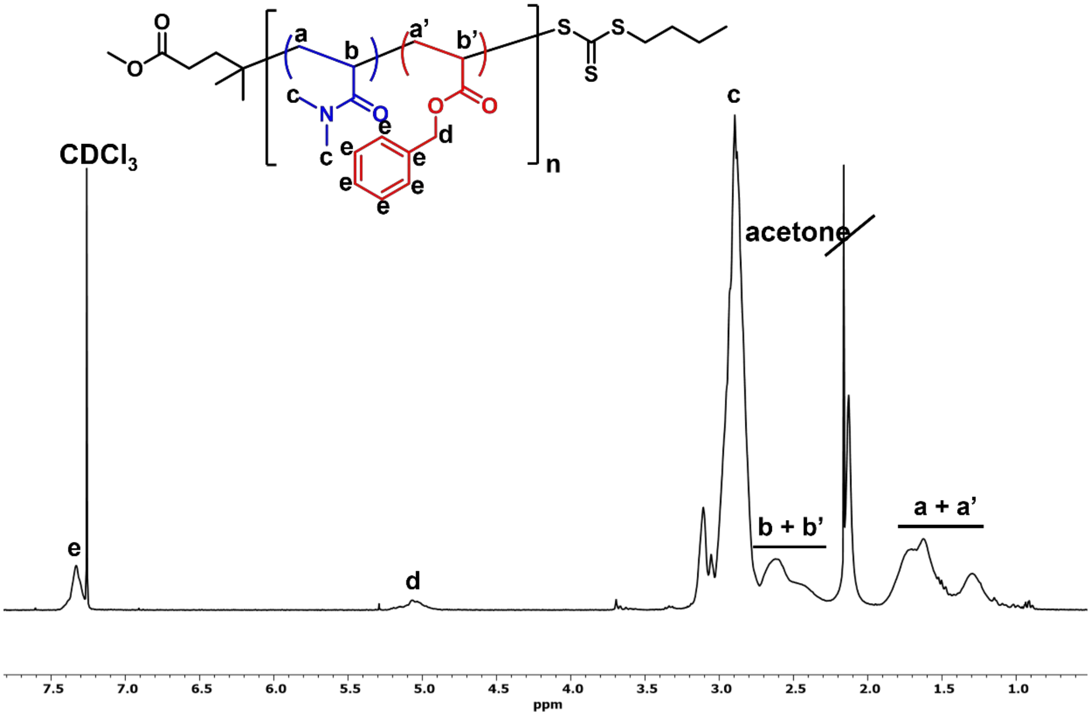

Figure S2. ^{1}H-NMR spectrum of P(DMA-*co*-BA)$_{82}$ in $CDCl_3$ (300 MHz).

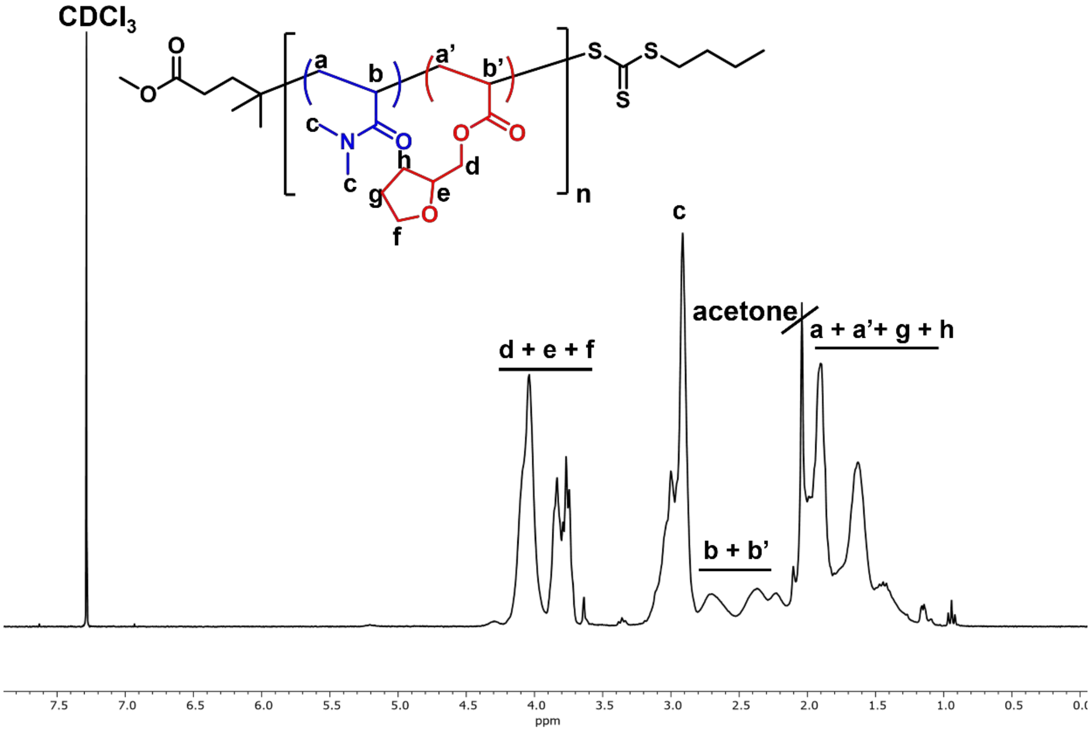

Figure S3. ^{1}H-NMR spectrum of P(DMA-*co*-THFA)$_{81}$ in $CDCl_3$ (300 MHz).

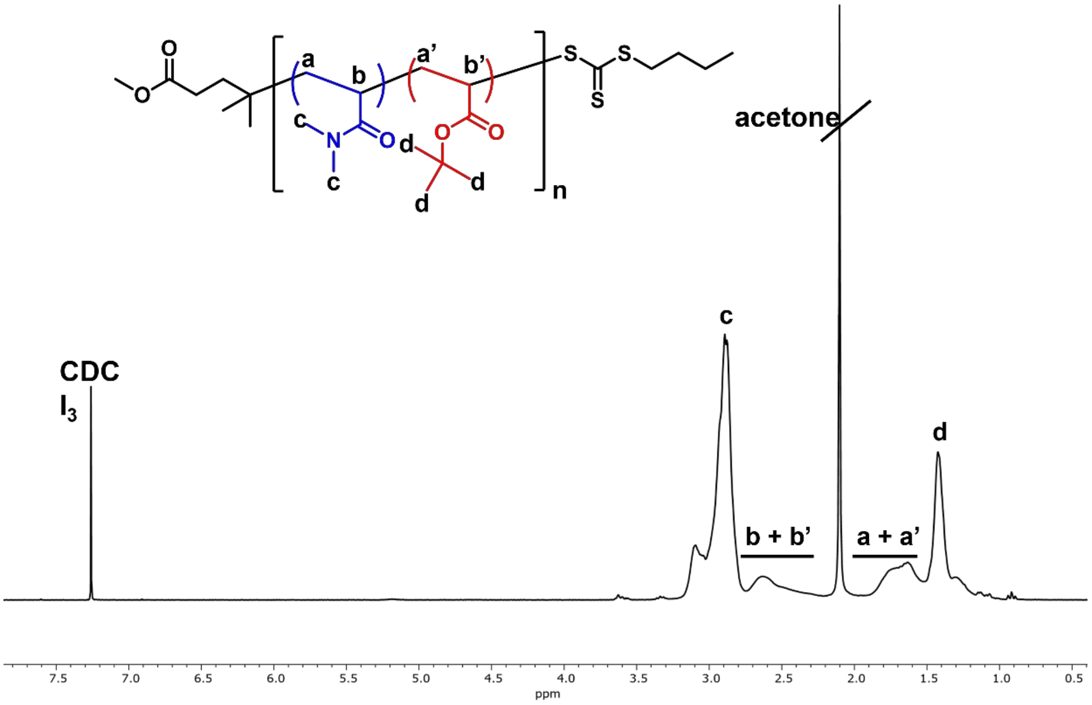

Figure S4. ^{1}H-NMR spectrum of P(DMA-*co*-*t*BuA)$_{84}$ in $CDCl_3$ (300 MHz).

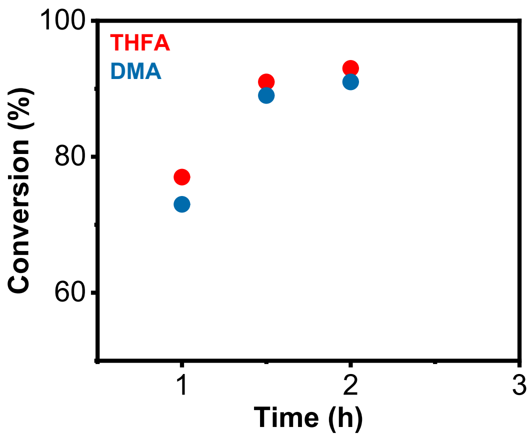

Figure S5. Conversion of THFA and DMA monomers for the 30% THFA system determined by ^{1}H NMR spectroscopy ($CDCl_3$, 300 MHz).

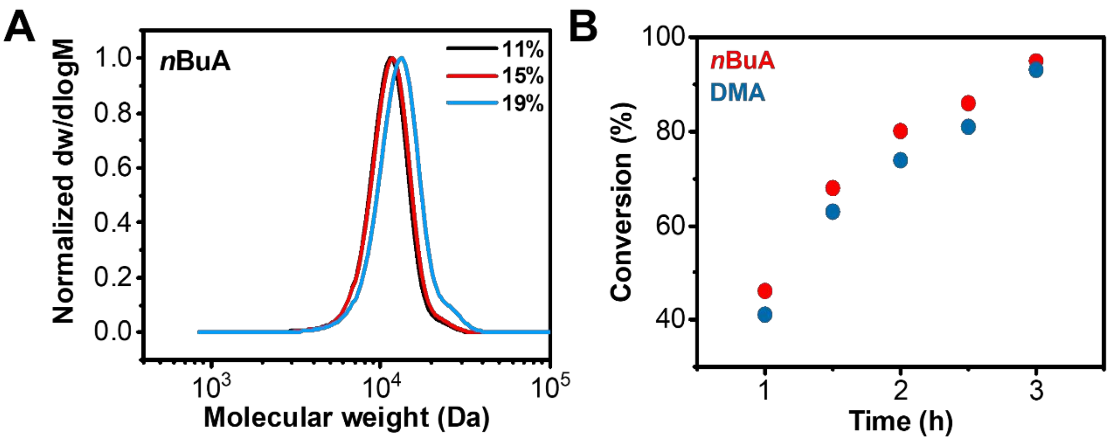

Figure S6. **(A)** Normalized molecular weight distributions for P(DMA-*co*-*n*BuA) copolymers determined by SEC ($CHCl_3$ + 0.5% NEt_3 as the eluent and calibrated against PMMA standards) using an RI detector. The legend shows the molar percentage of *n*BuA in the final copolymers. **(B)** Conversion of *n*BuA and DMA monomers for the 10% *n*BuA system determined by ^{1}H NMR spectroscopy ($CDCl_3$, 300 MHz).

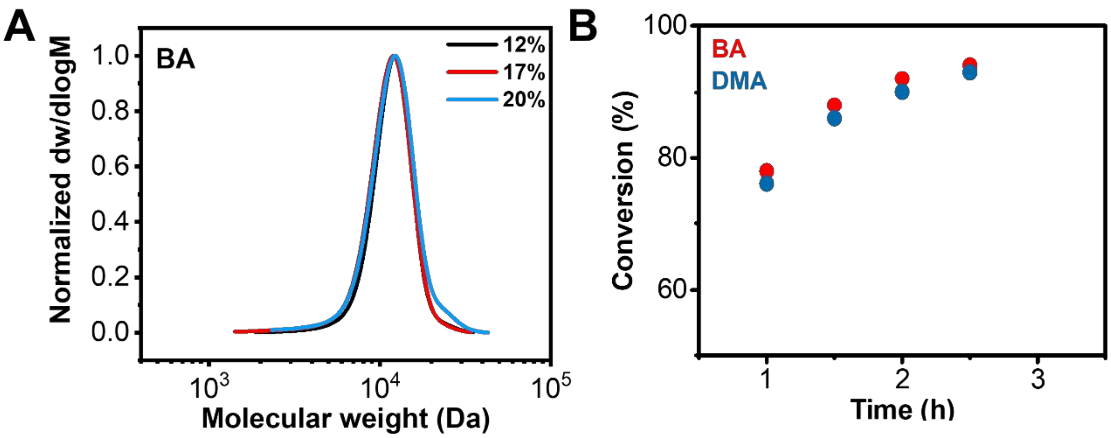

Figure S7. (A) Normalized molecular weight distributions for P(DMA-*co*-BA) copolymers determined by SEC ($CHCl_3$ + 0.5% NEt_3 as the eluent and calibrated against PMMA standards) using an RI detector. The legend shows the molar percentage of BA in the final copolymers. **(B)** Conversion of BA and DMA monomers for the 20% BA system determined by ^{1}H NMR spectroscopy ($CDCl_3$, 300 MHz).

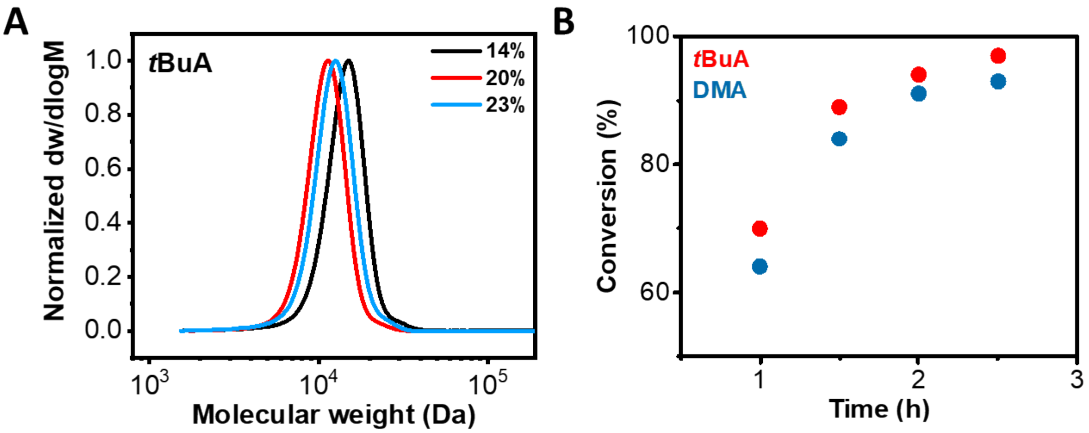

Figure S8. (A) Normalized molecular weight distributions for P(DMA-*co*-*t*BuA) copolymers determined by SEC ($CHCl_3$ + 0.5% NEt_3 as the eluent and calibrated against PMMA standards) using an RI detector. The legend shows the molar percentage of *t*BuA in the final copolymers. **(B)** Conversion of *t*BuA and DMA monomers for the 23% *t*BuA system determined by ^{1}H NMR spectroscopy ($CDCl_3$, 300 MHz).

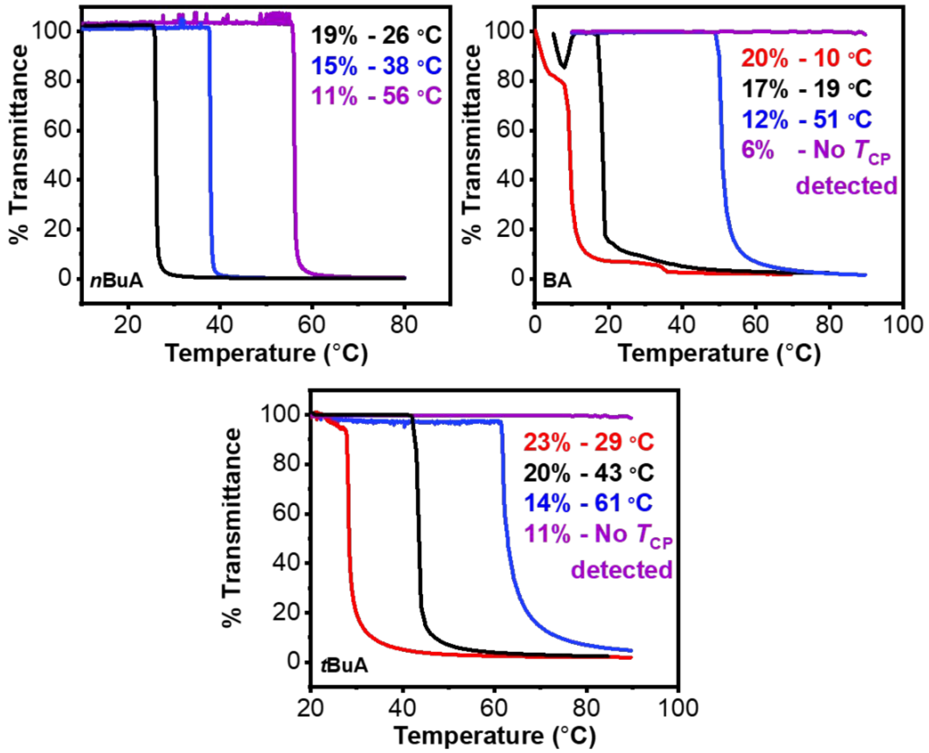

Figure S9. Turbidimetry analysis for P(DMA-*co*-RA) copolymers determined by UV-Vis spectrometry (0 °C to 93 °C, 1 °C min^{-1}, recorded at λ = 550 nm) at 10 mg ml^{-1}.

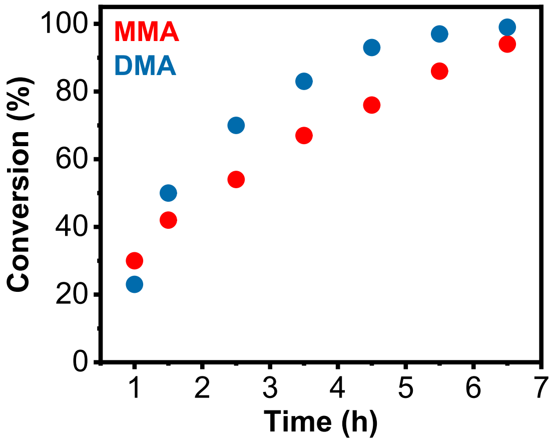

Figure S10. Conversion of MMA and DMA monomers for the 31% MMA system determined by ^{1}H NMR spectroscopy ($CDCl_3$, 300 MHz).

Table S2. T_{CP} of P(DMA-*co*-RA) copolymers determined by UV-Vis spectrometry.

Polymer[a]	T_{CP} from UV-Vis (°C)[b]	Polymer[a]	T_{CP} from UV-Vis (°C)[b]
**$P(DMA_{0.89}$-*co*-*n*$BuA_{0.11})$**	56	**$P(DMA_{0.79}$-co-$THFA_{0.21})$**	*soluble*
**$P(DMA_{0.85}$-*co*-*n*$BuA_{0.15})$**	38	**$P(DMA_{0.70}$-co-$THFA_{0.30})$**	58
**$P(DMA_{0.81}$-*co*-*n*$BuA_{0.19})$**	26	**$P(DMA_{0.60}$-co-$THFA_{0.40})$**	37
**$P(DMA_{0.94}$-*co*-$BA_{0.06})$**	*soluble*	**$P(DMA_{0.51}$-co-$THFA_{0.49})$**	27
**$P(DMA_{0.88}$-*co*-$BA_{0.12})$**	51	**$P(DMA_{0.42}$-co-$THFA_{0.58})$**	15
**$P(DMA_{0.83}$-*co*-$BA_{0.17})$**	19	**$P(DMA_{0.86}$-co-*t*$BuA_{0.14)}$**	61
**$P(DMA_{0.80}$-*co*-$BA_{0.20})$**	10	**$P(DMA_{0.80}$-co-*t*$BuA_{0.20})$**	43
		**$P(DMA_{0.77}$-co-*t*$BuA_{0.23})$**	29

[a] Aqueous solution of copolymers were prepared at 10 mg mL^{-1} in nanopure water.
[b] Data obtained from the second heating of the samples within the temperature range of 0 °C to 90 °C at a heating rate of 1 °C min^{-1}.

Table S3. Linear regression data for the P(DMA-*co*-RA) copolymers.

Polymer	Intercept	Slope
**P(DMA-*co*-*n*BuA)**	98	-3.8
**P(DMA-*co*-BA)**	117	-5.6
**P(DMA-*co*-THFA)**	101	-1.5
**P(DMA-*co*-*t*BuA)**	83	-1.9

References

1. Wang, X.; Shi, Y.; Graff, R. W.; Cao, X.; Gao, H., Synthesis of Hyperbranched Polymers with High Molecular Weight in the Homopolymerization of Polymerizable Trithiocarbonate Transfer Agent without Thermal Initiator. *Macromolecules* **2016,** *49* (17), 6471-6479.
2. *Materials Studio 2020*, Accelrys Software Inc.: San Diego, 2020.
3. Ghose, A. K.; Viswanadhan, V. N.; Wendoloski, J. J., Prediction of Hydrophobic (Lipophilic) Properties of Small Organic Molecules Using Fragmental Methods: An Analysis of ALOGP and CLOGP Methods. *J. Phys. Chem. A* **1998,** *102* (21), 3762-3772.
4. Min, K.; Cuiffi, J. D.; Mathers, R. T., Ranking environmental degradation trends of plastic marine debris based on physical properties and molecular structure. *Nat. Commun.* **2020,** *11* (1), 727.
5. Varlas, S.; Foster, J. C.; Arkinstall, L. A.; Jones, J. R.; Keogh, R.; Mathers, R. T.; O'Reilly, R. K., Predicting Monomers for Use in Aqueous Ring-Opening Metathesis Polymerization-Induced Self-Assembly. *ACS Macro Lett.* **2019,** *8* (4), 466-472.

6. Foster, J. C.; Varlas, S.; Couturaud, B.; Jones, J. R.; Keogh, R.; Mathers, R. T.; O'Reilly, R. K., Predicting Monomers for use in Polymerization Induced Self-Assembly. *Angew. Chem. Int. Ed.* **2018,** *57* (48), 15733-15737.
7. Dharmaratne, N. U.; Jouaneh, T. M. M.; Kiesewetter, M. K.; Mathers, R. T., Quantitative Measurements of Polymer Hydrophobicity Based on Functional Group Identity and Oligomer Length. *Macromolecules* **2018**.
8. Sun, H.; Jin, Z.; Yang, C. W.; Akkermans, R. L. C.; Robertson, S. H.; Spenley, N. A.; Miller, S.; Todd, S. M., COMPASS II: extended coverage for polymer and drug-like molecule databases. *J. Mol. Model.* **2016,** *22* (2).
